# Patients' Perceptions on the Performance of a Local Health System to Eliminate Leprosy, Paraná State, Brazil

**DOI:** 10.1371/journal.pntd.0003324

**Published:** 2014-11-20

**Authors:** Flávia Meneguetti Pieri, Michelle Mosna Touso, Ludmila Barbosa Bandeira Rodrigues, Mellina Yamamura, Ione Carvalho Pinto, Elma Mathias Dessunti, Juliane de Almeida Crispim, Antônio Carlos Vieira Ramos, Luiz Henrique Arroyo, Marcelino Santos Neto, Maria Concebida da Cunha Garcia, Marcela Paschoal Popolin, Tatiane Ramos dos Santos Silveira, Ricardo Alexandre Arcêncio

**Affiliations:** 1 Department of Nursing, Universidade Estadual de Londrina, Londrina, Paraná, Brazil; 2 Nursing and Public Health Graduate Program, University of São Paulo at Ribeirão Preto College of Nursing, Ribeirão Preto, São Paulo, Brazil; 3 Department of Medicine, Universidade Federal de Mato Grosso, Cuiabá, Mato Grosso, Brazil; 4 Department of Maternal-Infantile Nursing and Public Health, University of São Paulo at Ribeirão Preto College of Nursing, Ribeirão Preto, São Paulo, Brazil; 5 University of São Paulo at Ribeirão Preto College of Nursing, Ribeirão Preto, São Paulo, Brazil; 6 Center of Social Science, Health and Technology, Universidade Federal do Maranhão, Imperatriz, Maranhão, Brazil; University of California San Diego School of Medicine, United States of America

## Abstract

**Background:**

In Brazil, leprosy has been listed among the health priorities since 2006, in a plan known as the “Pact for life” (Pacto pela Vida). It is the sole country on the American continent that has not reached the global goal of disease elimination. Local health systems face many challenges to achieve this global goal. The study aimed to investigate how patients perceive the local health system's performance to eliminate leprosy and whether these perceptions differ in terms of the patients' income.

**Methodology/Principal Findings:**

A cross-sectional study was conducted in Londrina, State of Paraná, Brazil. Interviews were performed with the leprosy patients. The local health system was assessed through a structured and adapted tool, considering the domains judged as good quality of health care. The authors used univariate, bivariate and multivariate analyses. One hundred and nineteen patients were recruited for the study, 50.4% (60) of them were male, 54.0% (64) were between 42 and 65 years old and 66.3% (79) had finished elementary school. The results showed that patients used the Primary Health Care service near their place of residence but did not receive the leprosy diagnosis there. Important advances of this health system were verified for the elimination of leprosy, verifying protocols for good care delivery to the leprosy patients, but these services did not develop collective health actions and did not engage the patients' family members and community.

**Conclusions/Significance:**

The patients' difficulty was observed to have access to the diagnosis and treatment at health services near their homes. Leprosy care is provided at the specialized level, where the patients strongly bond with the teams. The care process is individual, with limited perspectives of integration among the health services for the purpose of case management and social mobilization of the community to the leprosy problem.

## Author Summary

Brazil still has not achieved the goal of leprosy elimination established by World Health Organization. The diagnosis and treatment of leprosy is easy and the country is striving to fully integrate leprosy services into existing general health services. Access to information, diagnosis and treatment with multidrug therapy (MDT) remain key elements in the strategy to eliminate the disease as a public health problem, defined as reaching a prevalence of less than one leprosy case per 10,000 population. Thus, this study aimed to investigate the performance of a local health system to eliminate leprosy through a cross-sectional study with leprosy patients. One hundred and nineteen patients were recruited for the study, 50.4% of them (60) were male, 54.0% (64) were between 42 and 65 years old and 66.3% (79) had finished elementary school. The health teams have invested only in the individual perspective and they do not coordinate the patients' care and, also, their practices are fragmented without perspectives of integration between different health services and social mobilization, an essential condition for the development of an effective and responsive system.

## Introduction

Iniquity in access to health services has been discussed among health authorities in developing countries, especially regarding poverty and neglected diseases, such as leprosy [1]. In recent years, in Brazil, the prevalence of this disease has progressively declined; nevertheless, this is the only country on the American continent that has not reached the global goal of disease elimination, with a detection rate of approximately 17.2 per 100,000 inhabitants [2].

Therefore, leprosy has been listed among the health priorities since 2006, in a plan known as the “Pact for life” (Pacto pela Vida) [3]. Local health systems face many challenges to achieve the global goal of leprosy elimination though. Most leprosy cases have been diagnosed late and this entails serious consequences for the individuals, due to the possibility of physical disabilities, negatively affecting self-care, work capacity and social relationships. [4], [5], [6], [7], [8].

It is also highlighted that only 1/3 of the patients are reported in the country, so that many continue transmitting the disease in the communities without being reached by local health systems [9]. Among the patients who get treated, it is estimated that a large majority do so irregularly or drop out, leading to drug-resistant bacilli, which aggravates the context of the disease in Brazil [10].

Although the country registers a drop in prevalence rates in recent years, it is far from achieving the elimination target of leprosy (<1 case per 10,000 population), unless the local health systems perform their functions as good as possible, changing their organizational and management logic [11].

The importance of a system focused on chronic conditions has been described in the literature, which strengthens prevention and health promotion actions [12], important measures to break the transmission chain of leprosy. Nevertheless, few studies intend to investigate the performance of local health systems from this more comprehensive perspective.

Despite conceptual disagreement among the authors, the term performance has been employed to express the extent to which the local health systems reach their objectives [13]. These objectives are in line with the values representations and needs of each population, so that each system will have a peculiar form of defining and producing its health actions [13]

Therefore, it is important to analyze how these local health systems have operated, within what logic and the extent of their evolution towards the elimination of leprosy. Studies on local health system performance can play a strategic role in the achievement of the elimination target, in terms of rethinking processes and triggering changes. Thus, the aim in this study was to investigate how patients perceive the local health system's performance to eliminate leprosy and whether these perceptions differ in terms of these patients' income.

## Methods

### Ethical statement

Ethical approval for the study was obtained from the University of São Paulo at Ribeirão Preto College of Nursing (Protocol number 08811212.0.0000.5393). All study participants signed a written consent form.

### Study design and place

A cross-sectional study [14] was carried out in Londrina, State of Paraná, Brazil. Londrina is located in the State of Paraná, Brazil, a hyperendemic area for leprosy. In 2012, its detection rate corresponded to 8.72 per 100,000 inhabitants and nearly one case per 100,000 inhabitants was diagnosed with grade 2 disability, which refers to the presence of a visible deformity or apparent physical damage and when sight is severely compromised [15]

The city is located in the South of Brazil, at a distance of 1,103.1 kilometers from Brasilia. The population amounted to 515,707 inhabitants, with a life expectancy of 71.37 years old in 2008 and a human development index of 0.824 [16]. The Family Health Program, an important program launched in Brazil to strengthen Primary Health Care (PHC), covers nearly 58% of the population [17]. Since 2009, the local health authorities have initiated a decentralization of leprosy control actions to the PHC.

### Population and sample

The study population consisted of 165 leprosy patients, identified through the Notifiable Diseases Information System (SINAN). For the study, the authors considered patients diagnosed from 01 January 2009 to 31 December 2012. The inclusion criteria were: patient with an address in the urban area of Londrina and who were 18 years old or older. The authors considered as exclusion criteria: patients who lived in rural areas, corresponding to the regions of the city beyond the urban perimeter [18] or who were not found at home after three visits by the researchers.

**Figure 1:**
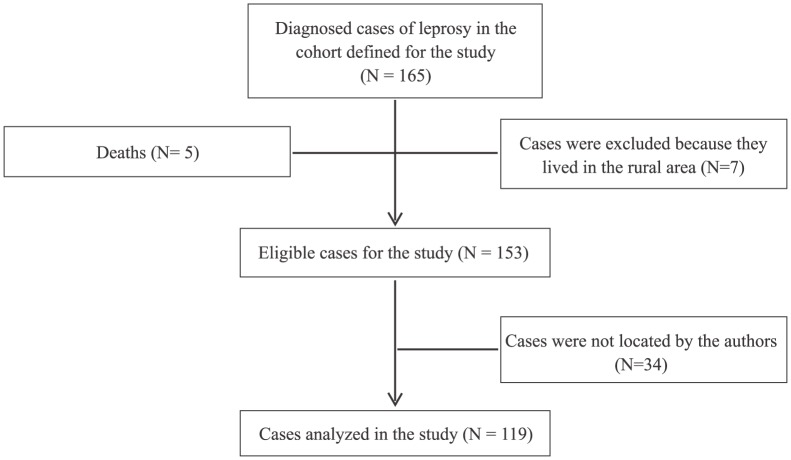
Flow chart of eligible and selected participants for the study, Londrina, Paraná, Brazil.

Figure 1 shows the numbers of individuals in each study stage and the eligible and analyzed participants.

### Data collection instrument and measures

The data was obtained through the application of a questionnaire adapted from the PHC Assessment Tool (PCATool) [19] and validated by Villa and Ruffino-Neto (2009) [20] to study the performance of health systems in the control of chronic transmissible and neglected diseases, such as tuberculosis, HIV and leprosy. The authors also collected sociodemographic data and patient characteristics and the type of health services sought at the onset of signs and symptoms. Participants answered a tool using a five-item Likert response scale: never true (1), somewhat true (2), true half of the times (3), mostly true (4), always true (5).

The tool was structured in two parts. The first investigated the participants' sociodemographic characteristics and the second was structured in nine attributes, being First contact, Access to the diagnosis, Access to treatment, Comprehensiveness of services, Longitudinality-relational, Coordination and Collaborative health actions, Family centeredness, Community orientation and Interpersonal communication. These domains have been proposed as assessment criteria to judge the quality of health care [13], [14]. The key definitions of the domains were based on the study developed by Haggerty et al. [21]. Table 1 presents the key definitions of each domain investigated and the number of items each domain contains.

**Table 1:** Domains and their key definitions of the tool selected to assess the performance of a local health system in the elimination of leprosy, Londrina, State of Paraná, Brazil (2013).

Id.	Domains	Key definitions**	Number of items	Scale
01	First contact	The ease patients in need of care obtained.	04	Likert
02	Access to diagnosis	The resources employed to diagnose the leprosy early, considering the protocols established by the Ministry of Health.	09	
03	Access to treatment	The resources used to ensure the regular therapy of the Leprosy patients and their adherence.	10	
04	Comprehensiveness of services	A full range of services to meet patients' health care needs. This includes health promotion, prevention of the disease, social support and rehabilitation.	11	
05	Longitudinality-relational	A relationship established between a patient and health care professionals, resulting in accumulated knowledge of the patient and care consistent with the patient's needs	10	
06	Coordination and collaborative health actions	The delivery of services by different health care professionals in a timely and complementary manner, so that care is connected and coherent. It is also considered in this domain whether health care professionals used past care information to support their decision about the best care of leprosy patients.	11	
07	Family centeredness	The extent to which the health care professionals consider the family, understand its influence on a person's health and engage it as a partner in ongoing health care	09	
08	Community orientation	Social mobilization of the community to discuss the leprosy problem.	05	
09	Interpersonal Communication	The ability of the health care professionals to elicit and understand patients concerns and engage them in the decision making about the care and patients' satisfaction with this process.	07	

**Modified from Haggerty et al. (2007).

### Data collection

Data collection was conducted between June and September 2013. First, authors contacted the Epidemiological Health Surveillance of the Londrina City Health Secretariat to identify patients diagnosed with leprosy and their addresses during the study period.

The authors visited all health care services (HCS) where the patients were being followed to introduce the study to the coordinators and health professionals. On that occasion, all addresses and phones of patients were updated. Subsequently, the patients were invited by telephone and, when they did not possess a phone number or had a telephone but the authors were not able to contact them, the researchers visited their home to invite them to the study. If they consented, a convenient time and place were set for the interviews.

### Data analysis

Two persons typed the data independently, after which both files were confronted to check for inconsistencies, using the software Statistica version 12.0.

To assess the performance of the local health system to eliminate leprosy, the steps defined in other studies for performance assessment were followed [19].

Initially, univariate analysis was carried out with description of position (mean and median) and dispersion measures (standard deviation) of the study variables. Then, the mean score of each indicator in the tool was obtained, considering the sum of scores for each item divided by the number of participants. It also was computed a 95% confidence interval for the mean. The attributes were constructed based on the mean item scores. The following criteria were adopted: indicator below 3 unsatisfactory; between 3 and 4 regular; and 4 or more satisfactory.

Bivariate analyses were conducted, comparing leprosy patient groups with different level of incomes in relation to the attributes investigated. To stratify the groups regarding the income, the researchers considered the quartiles low, medium and high. Subsequently, the authors used one-way ANOVA and Kruskal-Wallis, the latter when the criteria of normality and homoscedasticity were not confirmed [22]. A two-sided p-value of ≤0.05 was defined as statistically significant.

## Results

One hundred and nineteen subjects participated in the study. None of the subjects contacted refused to participate. Twenty two percent of the patients (26) were classified as paucibacillary and 78.0% (93) as multibacillary. Table 2 shows the participants' sociodemographic characteristics. There is a balance among the sexes in terms of patients affected by leprosy. The participants' ages varied between 42 and 65 years old and 66.4% (79) had finished primary school. In addition, 50.4% (69) patients were married and most of them gained between 1.2 and 3.3 minimum wages (MW) per month.

**Table 2:** Sociodemographic characteristics of leprosy patients selected for the study, Londrina, State of Paraná, Brazil (2013).

Variables	N	%
**Sex**		
Female	59	49.6
Male	60	50.4
**Age**		
<42	29	24.4
42–65	64	53.8
>65	26	21.8
**Education**		
None	10	8.4
Primary education	79	66.4
Secondary education	24	20.2
Higher education	6	5.0
**Marital situation**		
Single	14	11.76
Married	60	50.42
Fixed partner	14	11.76
Divorced	14	11.76
Widowed	17	14.3
**Income** **		
<800	17	14.3
800–2220	73	61.3
>2200	29	24.4
**Profession**		
Unemployed	4	3.4
Retired	36	30.2
Disability retired/disease aid	11	9.2
Housewife	12	10.1
Other	56	47.1
**Housing**		
Self-owned	96	80.7
Hired	20	16.8
Institution (asylum, shelter, others)	2	1.7
Não tem moradia (morador de rua)	1	0.8
**Housing type**		
Concrete	107	89.9
Wood	8	6.7
Mixed (concrete and wood)	4	3.4
**People living in the same house**		
≤3	69	58
>3	50	42.0

**Minimum Wage at the time equivalent to U$ 304.00.

Considering the employment status, 47.1% (56) were employed, most of them self-employed; 30.2% (36) were retired and 9.2% (11) disability retired because of the disease. Regarding the patients' housing conditions, most of them lived in their own house, made of concrete.

Table 3 displays the items related to the domains investigated to assess the performance of the local health system. Regarding the first contact domain, it was observed that leprosy patients eventually seek the PHC for preventive actions and also to solve their health problems. It can also be identified that the patients do not access the emergency services when needed.

**Table 3:** Performance of a local health system to eliminate leprosy according to the domains first contact, access to diagnosis and treatment, Londrina, State of Paraná, Brazil (2013).

Domains	Variables	Mean (±SD)	95% CI
First Contact	The patients sought the emergency health services when they had some health problem	4.4 (0.9)	4.2–4.6
	The patients sought preventive care in the Primary Health Care service	3.8 (1.4)	3.5–4.1
	The patients sought Primary Health Care when they had some health problem	3.7 (1.7)	3.4–4.0
	The patients sought Primary Health Care to be referenced to the specialist professionals	2.2 (1.5)	1.9–2.4
	General	3.5 (0.6)	3.4–3.6
Access to diagnosis	The ease to schedule an appointment by telephone	4.4 (1.1)	4.2–4.7
	The ease to obtain the information needed about the health problem	4.3 (1.4)	4.0–4.5
	The ease to reach the health care services	3.8 (1.5)	3.5–4.1
	The patients sought Primary Health Care at the onset of the leprosy symptoms.	3.5 (1.7)	3.2–3.8
	The patients had agenda problems due to medical appointments scheduled by health care services	3.1 (1.7)	2.8–3.4
	The ease to obtain assistance and be diagnosed with leprosy	3.0 (1.7)	2.7–3.4
	Agility in the medical appointment within 24 hours when the patient demanded the health care services for the first time	1.9 (1.6)	1.7–2.2
	The patients have used motorized transport to attend the health care services	1.3 (1.0)	1.2–1.5
	The patients had transportation costs to seek the health care services	1.2 (0.8)	1.1–1.3
	General	3.0 (0.7)	2.8–3.1
Access to treatment	Uninterrupted supply of medicine	4,9 (0.5)	4.7–4.9
	The ease to schedule consultation appointments during the patients' treatment	4.8 (0.7)	4.7–4.9
	The ease to access to information about the leprosy, duration of the therapy, side effects, transmission mode, among others.	4.6 (1.0)	4.4–4.8
	Impairment of personal agenda due to consultation appointments scheduled by the health care services (not flexible)	2.8 (1.7)	2.5-3.1
	The ease to get assistance when the patients have a problem related to the side effects of medication or other intercurrences	2.2 (1.6)	1.9–2.5
	Waiting time for the medical appointments	2.2 (1.4)	2.0–2.5
	Frequency of home visits	2.2 (1.3)	1.9–2.4
	Geographical accessibility to the health care services	1.3 (0.9)	1.1–1.5
	Expenditures with the treatment relating to transport and other thinks	1.3 (0.9)	1.1–1.5
	The leprosy treatment is developed through the Primary Health Care service near the patients' homes.	1.2 (0.9)	1.1–1.4
	General	3.0 (0.4)	2.9–3.1

As to the access to diagnosis (Table 3), it was verified that the patients had to visit the health services thrice before the leprosy diagnosis was reached. The patients had easy access to schedule their medical appointments by telephone; agility in getting the consultations was not verified though, showing unsatisfactory results.

The results also revealed that patients have transportation costs before the diagnosis and during the treatment because the HCS are distant from their homes and the treatment is not supported by the PHC. It was observed that health care professionals have oriented the patients about the leprosy, its signs and symptoms and mode of transmission, among others, but that these professionals do not visit the patients' home and, in case of problems, such as side effects of the medication, the patients do not get a medical appointment within 24 hours.

Table 4 shows the items related to the longitudinality-relational and interpersonal relationship domains. In relation to the longitudinality-relational domain, it was observed that health care professionals register all issues of the patients in the charts and understand their doubts and concerns. The HCP clearly respond to these issues, but do not dialogue about other subjects beyond the disease. Most item scores are satisfactory in this domain, except for the orientation about the types of medication used in the patients' treatment and social support offered by the HCP.

**Table 4:** Performance of a local health system to eliminate leprosy according to the domains longitudinality-relational and interpersonal communication, Londrina, State of Paraná, Brazil (2013).

Domains	Variables	Mean (±SD)	95% CI
Longitudinality-relational	The issues of the patients are registered by health care professionals in the chart or other register system	4.8 (0.8)	4.7–5.0
	Enough time for the patients to talk about their doubts and concerned in the medical appointments	4.8 (0.8)	4.6–4.9
	The health care professionals responded clearly to the patients' issues	4.8 (0.8)	4.6–4.9
	The Health Care professionals understood the patients' doubts and concerns	4.8 (0.5)	4.8–5.0
	Patients were serviced by the same health care professionals	4.7 (0.7)	4.6–4.8
	Patients had access to the health care professionals who clarified their doubts about leprosy	4.7 (0.7)	4.6–4.9
	Patients' opinion about the health care professionals who provided their care.	4.6 (0.6)	4.5–4.8
	The health care professionals investigated other medicines used by the patients beyond those considered for the leprosy treatment	3.8 (1.5)	3.6–4.1
	The patients were oriented about the treatment period and types of drugs considered for their therapy	3.6 (1.5)	3.3–3.9
	The Health Care professionals inquired the patient about other health problems beyond the leprosy disease	2.6 (1.7)	2.2–2.9
	Health Care professionals provided social support to the patients, such as food basket, transportation vouchers, food tickets and others social benefits	1.1 (0.3)	1.1–1.2
	General	4.0 (0.5)	4.0–4.1
Interpersonal communication	Assessment of the patients about the help offered by the health care professionals	4.9 (0.3)	4.9–5.0
	Welcoming of the patients by the health care professionals	4.8 (0.8)	4.6–4.9
	Relationship between the health care professionals and the community.	4.4 (1.0)	4.2–4.6
	The patients wished to change to the other health care services because of the unsatisfactory interpersonal relationship with the health care professionals.	4.3 (1.3)	4.1–4.5
	The patients recommended the health care services to their community	4.0 (1.5)	3.7–4.2
	Availability of the health care professionals to provide the care on work days.	3.7 (1.3)	3.4–3.9
	General	4.2 (0.7)	4.1–4.3

The results also show that patients positively assessed the help offered by HCP and felt welcomed. The patients also refer that the relationship between the HCP and community was positive and well established. It was observed that the HCP were not always available to the leprosy patients on workdays.

Table 5 presents the domains associated to the comprehensiveness of services provided by the HCS. It was observed that all patients received their medication, without any shortages. These aspects were considered satisfactory. There were regular medical appointments for the leprosy patients but the BCG vaccine was not available to all patients' contacts. Although most of the participants were multibacillary, the microscopy was produced insufficiently. It was also observed that HCP did not visit the patients' homes and that care is provided in the HCS only. Also, the HCP did not develop health promotion actions with the patients.

**Table 5:** Performance of a local health system to eliminate leprosy according to the domains comprehensiveness of services and coordination and collaborative health actions, Londrina, State of Paraná, Brazil (2013).

Domains	Variables	Mean (±SD)	95% CI
Comprehensiveness of services	Delivery of multidrugs	5.0 (0.4)	4.9–5.0
	The medicine intake was monitored by the health care Professionals at least once per month	4.6 (1.1)	4.4–4.8
	The patients had regular medical appointments	4.1 (1.1)	3.9–4.3
	The health care professionals provided physical, dermatological and neurological examinations	3.5 (1.1)	3.3–3.7
	The health care professionals provided the BCG vaccine	2.8 (1.8)	2.5–3.1
	The health care professionals performed the Lepromin Skin Test	2.8 (1.3)	2.6–3.1
	The health care professional offered the microscopy	2.6 (1.2)	2.4–2.8
	The health care professional offered the biopsy of the lesion and stain	2.3 (1.2)	2.3–2.6
	Health care professionals visited the patients' homes during the treatment to search their contacts	2.2 (1.3)	1.9–2.4
	Health care professionals visited the patients' homes during the treatment because of other reasons beyond the disease	1.9 (1.3)	1.7–2.1
	Health promotion actions were offered to the patients and their families	1.8 (1.3)	1.6–2.1
	The patients participated in the leprosy patient groups to share their experiences and learn from them	1.3 (0.8)	1.2–1.5
	General	2.7 (0.5)	2.6–2.8
Coordination and collaborative health actions	The health care professionals used the patients' charts during the medical appointments	4.6 (1.0)	4.4–4.8
	The examination results of the patients were available to all health care professionals who provided their care	4.5(1.1)	4.3–4.7
	The patients received the registered orientations that medical appointments with the specialist professionals were confirmed and the location, when these appointments were necessary.	4.5 (1.2)	4.2–4.7
	The health care professionals helped the patients to obtain medical appointments with other specialist professionals	4.5 (1.1)	4.3–4.7
	The health care professionals advised the patients early about their return appointments	4.4 (1.33)	4.5–4.6
	The patients' referral decision was shared with them	4.2 (1.3)	3.9–4.4
	The patients were referenced to the medical specialists when necessary.	4.1 (1.4)	3.9–4.4
	The referral process to the specialist professionals was well established in the health care services	3.3 (1.7)	3.0–3.6
	There was a communication flow between the health care services where the patients were being followed to treat leprosy and specialized care services they requested.	1.8 (1.4)	1.5–2.0
	The health care professionals were concerned with the quality of the care provided by the specialist professionals.	1.6 (1.3)	1.4–1.8
	The health care professionals who are providing care to the patients shared opinions and discussed their cases with specialist medicals of the other health care services	1.5 (1.3)	1.3–1.8
	General	3.5 (0.7)	3.4–3.7

In relation to the coordination of care, Table 5 shows that HCP used the chart to register the issues of the patients and help them to obtain a medical appointment with the specialist professionals, in case of need. Nevertheless, there was no communication flow between these services and the health unit where the patients were being followed to treat leprosy. It was verified in this table that HCS did not share opinions with the specialist professionals about the patients' health of the patients and did not inquire of the patients about the quality of care these providers offered.

Table 6 shows that the health care professionals did not provide care to the patients' family members, nor did they offer the BCG vaccine or investigated the living conditions of all patients' family members. There is no surveillance control of the patients' contacts and the HCP did not engage their families in the care. The family members were not taught about the leprosy disease, its signals and symptoms, transmission mode, therapy, among others.

**Table 6:** Performance of a local health system to eliminate leprosy according to the domains family centeredness and community orientation, Londrina, State of Paraná, Brazil (2013).

Domains	Variables	Mean (±SD)	95% CI
Family centeredness	The health care professionals knew all patients' family members.	3.9 (1.6)	3.6–4.1
	The health care professional provided BCG vaccine to all patients' family members	3.2 (1.9)	2.9–3.5
	The health care professionals searched to investigate the living conditions of all patients' family members	3.1 (1.8)	2.7–3.4
	The health care professionals requested information about all patients' family members	2.4 (1.5)	2.1–2.7
	The health care professionals searched skin lesions and stains among all patients' family members	2.4 (1.3)	2.1–2.6
	The health care professionals provided surveillance and prevention actions for all patients' family members	2.2 (1.3)	2.0–2.4
	The health care professionals taught the patients' family about the disease, its signs and symptoms, mode of transmission, therapy, among others.	1.8 (1.2)	1.6–2.0
	The health care professionals shared their opinions and decisions about the patients 'treatment with their family members	1.8 (1.1)	1.6–2.0
	General	2.5 (0.9)	2.3–2.6
Community orientation	The health care professionals assessed the community's opinions about the impact of their actions and responsibility.	3.4 (1.3)	3.1–3.6
	There were campaigns or advertisements in the patients' community to raise the social awareness about the problem of leprosy	2.3 (1.4)	2.0–2.5
	There was active case finding of the disease in the community	1.3 (0.9)	1.2–1.5
	There were campaigns to mobilize the community about the importance of the physical skin exam and neurological sensitivity test.	1.3 (0.8)	1.1–1.4
	There was social mobilization for the elimination of leprosy in the patients' communities.	1.1 (0.5)	1.0–1.2
	General	1.9 (0.6)	1.8–2.0

In relation to collective actions, the results show that HCP did not engage the community in discussions about the problem of leprosy; there was no social mobilization to raise the community's awareness regarding the problem. Active case finding was another issue observed in this study.

In Table 7, when the income groups were compared, statistically significant differences were observed in relation to the first contact; the high-income group evaluated this domain more unsatisfactory than the low and medium income groups (p = 0.002). With regard to access to diagnosis, the low-income group assessed this domain as more unsatisfactory than the medium and income groups (p = 0.03). Concerning the comprehensiveness of services, it was also observed that the high-income group related more unsatisfactory values when compared with the low and medium income groups (p = 0.03).

**Table 7:** Assessment of the domains by the leprosy patients according to their income stratus, Londrina, State of Paraná, Brazil (2013).

Domains	Low Income		Medium income		High Income		P value
	(N = 17)		(N = 73)		(N = 29)		
	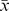 (±SD)	95% CI	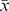 (±SD)	95% CI	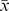 (±SD)	95% CI	
First Contact	2.9 (0.7)	2.6–3.3	3.0 (0.6)	2.8–3.1	2.4 (0.7)	2.2–2.7	<0.002**
Access to diagnosis	2.6 (0.6)	2.3–2.9	3.1 (0.8)	2.9–3.3	2.9 (0.6)	2.7–3.2	0.03**
Access to treatment	2.7 (0.4)	2.5–3.0	3.0 (0.5)	3.0–3.1	3.0 (0.4)	2.8–3.1	0.06
Longitudinality-relational	4.0 (0.5)	3.8–4.2	4.1 (0.4)	4.0–4.2	3.9 (0.6)	3.7–4.1	0.13
Comprehensiveness of services	2.8 (0.6)	2.6–3.1	2.8 (0.5)	2.7–2.9	2.5 (0.3)	2.4–2.7	0.03**
Coordination and collaborative health actions	3.4 (0.6)	3.2–3.8	3.6 (0.7)	3.4–3.8	3.4 (0.6)	3.1–3.6	0.33
Family centeredness	2.5 (0.9)	2.1–3.0	2.5 (1.0)	2.3–2.8	2.2 (0.8)	1.9–2.6	0.33
Community orientation	1.7 (0.5)	1.5–2.0	1.9 (0.6)	1.8–2.0	1.8 (0.5)	1.8–2.0	0.68
Interpersonal Communication	4.2 (0,8)	3.8–4.6	4.2 (0.7)	4.1–4.4	4.2 (0.7)	4.0–4.5	0.99

**p-value is statistically significant.

## Discussion

The study aimed to investigate how the patients perceive the system's performance to eliminate leprosy. As observed, globally, this performance has not been satisfactory, as leprosy is diagnosed in a late stage and not close to the patient's place of residence. It is highlighted that, although the patients turned to the Primary Health Care level when the symptoms started, that is not where the leprosy was diagnosed, which has entailed costs for the patients. At the specialized services where the patients were monitored, they satisfactorily assessed the relation with the health professionals at those services, but these are not articulated with the other care management and coordination services.

Another relevant result is the lack of social support for the patients, without the supply of basic food packages, food tickets, transportation, among other benefits, which are common in other public social programs in Brazil [11]. This system has not prioritized the active search for suspected cases in the community either, nor social mobilization activities with regard to the leprosy problem. Another objective of the study was to identify if the patients' perceptions varied with their income, showing that, with regard to the attributes entry door, access to the diagnosis and list of services, the patients with the highest income assessed these dimensions more negatively.

As regards the profile of the study participants, their sociodemographic characteristics are similar to the results found in the study by Hacker et al [23] regarding the absence of gender differences, with a slight predominance of male patients. The research findings also confirmed the patient profile described in the literature concerning advanced age, low education, own home and informal economic activity [24]–[28];

With respect to the access to the leprosy diagnosis, the patients turn to the units located near their place of residence, but that they did not get their diagnosis at these services, in line with data by Arantes et al [29]. Concerning the Longitudinality-relational dimension, satisfactory results were found for the referral centers, which indicate a good relation between the subjects and health professionals. These findings raise an important discussion about the quality of leprosy care at specialized services which, perhaps due to the establishment of protocols or clinical guidelines, are able to trigger a favorable ambience for bonding, free from discrimination, prejudice and stigma [30].

When considering the attribute Comprehensiveness of services, it was verified that the supply of some technologies for diagnosis and case monitoring is restricted, such as the supply of the BCG vaccine, Mitsuda test and dermato-neurologic review.

According to CDC recommendations [31], surveillance actions should mainly be focused on the patient's intra-domestic contacts, as they people are at a greater risk of being infected than the general population. These cases should be submitted to the dermato-neurologic review and receive the BCG vaccine, which increases the organism's resistance, mainly to the multibacillary forms of the disease. Nevertheless, the study results show weaknesses in the development of these actions, due to the lack of material, and also because the health services do not consider it a priority action.

As regards the collective actions, the results revealed that the health systems have not accomplished interventions in which the community and family are considered in the discussions about leprosy, whether through groups or other work methods, although health education and physical damage prevention and reduction are recommended in the National Plan for the Elimination of Leprosy (PNEH) as fundamental actions for disease control [32]. According to the authors [33], educative activities should become a part of the service routine, in order to disseminate appropriate information and sensitize the community about the disease.

The results also indicated weaknesses in the care coordination, especially in terms of counter-referral and articulation with other specialties for the purpose of case management. Coordination is an important attribute for the organization of the health production process, as this is a structuring principle in the transformation process of Primary Health Care from the perspective of service systems [34]. Without effective coordination, longitudinality loses its potential, comprehensiveness is hampered and the first contact turns into a merely administrative act [35]. In this conjuncture, it is considered that there are few services in Brazil that have been able to advance in terms of integrated health systems or Health Care Networks (HCN) coordinated by Primary Health Care (PHC) [36].

Another attribute assessed refers to the families' participation in care production for the patients, in which weaknesses were identified with regard to the surveillance of domestic contacts, orientations to the families about the disease, its treatment, clarification of other health problems and the application of the BCG vaccine to domestic contacts. The family's role is extremely important, serving as a support network that contributes to the patient's physical and mental balance [37]. When the family does not feel sufficiently informed about the disease, however, negative reactions may be triggered in the care process, besides contributing to the abandonment of treatment [38].

The findings regarding the orientations to the communities affected by the disease permit highlighting the importance of moving beyond the formal teaching spaces, such as knowledge transmission, to a space that accepts pedagogical practices that encourage critical reflections in the community and permit the attribution of a new meaning to the reality [39]. Therefore, orientations to the community need to be provided in a dialogic, emancipating, participatory and creative perspective, so as to contribute to the subjects' autonomy regarding their condition as subjects with rights and authors of their health and disease trajectory [40]. The results indicated a strong relation in the user's perception of the health professional, which may be related to the fact that the care process goes beyond technical competency, encompassing the interpersonal and humanistic aspects of the professional-patient relation. In that context, the health professionals should pay attention to the complaints and propose solutions in combination with the patient, establishing a relation based on welcoming and humanization [41].

When the health systems' performance attributes were verified in relation to patient groups with different income levels, it was observed that high-income groups assessed the entry door as less satisfactory than the low-income groups; the same was true for the comprehensiveness of services attribute.

These results raise the question about the social representation of the Unified Health System (SUS) for segment data about socially disadvantaged populations. In the study by Sorj and Martuccelli [42], the challenge is raised to establish a social protection model in Latin America that rescues the population in its citizenship and state of right. In that sense, the health production model needs to be considered, as well as the extent to which it is contributing to the construction of a universal and fair system.

Also according to the results, the decentralization has not culminated in the expanded access to Primary Health Care services in the local health system investigated. The different hypotheses to explain this result include the difficulty to conform to a new care model that is legitimized by the population as well as the health managers. Brazil has been a stage for disputes among different care models, especially the physician-centered model, which remains very strong [11]. Hence, the study results contain characteristics of this model centered on acute conditions and the disease. Nevertheless, the literature has shown more solid models that can achieve better health results [12].

As potentials, the originality of the research is emphasized, as no other study was found that assessed leprosy care in view of the PHC attributes. In addition, the study can serve as an important tool for managers to define local health policies for the elimination of leprosy and the reorganization of care services under the coordination of primary care professionals in the city under study. In line with some authors, a performance study should neither be an end in itself nor be forwarded as a strictly academic exercise, but should be focused on driving the development of health policies, strategies and programs, which was the direction taken in this study.

Based on the results, it can be concluded that it is important to advance in the reorganization of the health services, to establish communication protocols among the different professionals working in these systems, and to invest in and value PHC, as investments at this care level come with a lower cost and, when well structured and with good problem-solving ability, they can promote a balance between the improvement of the population's health and equity in the distribution of resources [34]. Using PHC as a partner can be an interesting measure to reduce the patients' expenses and involve the community in the treatment of leprosy.

### Study limitations

The study limitations refer to the memory bias, in which many patients may not have remembered facts or occurrences, due to the time passed since the end of treatment. In addition, the use of an adapted instrument for leprosy should be highlighted, which may cause an information bias, and the number of losses, as many patients were not located due to a changed or non-existent address.

## Supporting Information

Checklist S1 STROBE checklist.
(DOCX)
