# Supplementary material for: Patients' Perceptions on the Performance of a Local Health System to Eliminate Leprosy, Paraná State, Brazil
Source: PLoS Negl Trop Dis. 2014 Nov 20;8(11):e3324. doi: 10.1371/journal.pntd.0003324 (PMC4238994; doi:10.1371/journal.pntd.0003324)
Supplement: Checklist S1 — STROBE checklist. (DOCX) [file pntd.0003324.s001.docx]

STROBE Statement—checklist of items that should be included in reports of observational studies

|  | Item No. | Recommendation | Page  No. | Relevant text from manuscript |
| --- | --- | --- | --- | --- |
| **Title and abstract** | 1 | (*a*) Indicate the study’s design with a commonly used term in the title or the abstract | 2 | “A cross-sectional study was conducted in Londrina, State of Paraná, Brazil.” |
|  |  | (*b*) Provide in the abstract an informative and balanced summary of what was done and what was found | 2 | “The study aimed to investigate how patients perceive the local health system’s performance to eliminate leprosy and whether these perceptions differ in terms of the patients’ income”. “The patients’ difficulty was observed to have access to the diagnosis and treatment at health services near their homes”. |
| Introduction | | | |  |
| Background/rationale | 2 | Explain the scientific background and rationale for the investigation being reported | 3 | “The importance of a system focused on chronic conditions has been described in the literature, which strengthens prevention and health promotion actions, important measures to break the transmission chain of leprosy. Nevertheless, few studies intend to investigate the performance of local health systems from this more comprehensive perspective.” |
| Objectives | 3 | State specific objectives, including any prespecified hypotheses | 4 | “…the aim in this study was to investigate how patients perceive the local health system’s performance to eliminate leprosy and whether these perceptions differ in terms of these patients’ income.” |
| Methods | | | |  |
| Study design | 4 | Present key elements of study design early in the paper | 4 | “A cross-sectional study was carried out in Londrina, State of Paraná, Brazil.” |
| Setting | 5 | Describe the setting, locations, and relevant dates, including periods of recruitment, exposure, follow-up, and data collection | 4/5 | “The study population consisted of 165 leprosy patients”  “For the study, the authors considered patients diagnosed from 01 January 2009 to 31 December 2012.”  “Data collection was conducted between June and September 2013. First, authors contacted the Epidemiological Health Surveillance of the Londrina City Health Secretariat to identify patients diagnosed with leprosy and their addresses during the study period.” |
| Participants | 6 | (*a*) *Cohort study*—Give the eligibility criteria, and the sources and methods of selection of participants. Describe methods of follow-up  *Case-control study*—Give the eligibility criteria, and the sources and methods of case ascertainment and control selection. Give the rationale for the choice of cases and controls  *Cross-sectional study*—Give the eligibility criteria, and the sources and methods of selection of participants | 4 | “The inclusion criteria were: patient with an address in the urban area of Londrina and who were 18 years old or older. The authors considered as exclusion criteria: patients who lived in rural areas, corresponding to the regions of the city beyond the urban perimeter or who were not found at home after three visits by the researchers.” |
|  |  | (*b*) *Cohort study*—For matched studies, give matching criteria and number of exposed and unexposed  *Case-control study*—For matched studies, give matching criteria and the number of controls per case |  |  |
| Variables | 7 | Clearly define all outcomes, exposures, predictors, potential confounders, and effect modifiers. Give diagnostic criteria, if applicable | 4/5 | “The inclusion criteria were: patient with an address in the urban area of Londrina and who were 18 years old or older. The authors considered as exclusion criteria: patients who lived in rural areas, corresponding to the regions of the city beyond the urban perimeter [18] or who were not found at home after three visits by the researchers.”  “The data was obtained through the application of a questionnaire adapted from the PHC Assessment Tool (PCATool) and validated by Villa and Ruffino-Neto (2009) to study the performance of health systems in the control of chronic transmissible and neglected diseases, such as tuberculosis, HIV and leprosy. The authors also collected sociodemographic data and patient characteristics and the type of health services sought at the onset of signs and symptoms. Participants answered a tool using a five-item Likert response scale: never true (1), somewhat true (2), true half of the times (3), mostly true (4), always true (5).  The tool was structured in two parts. The first investigated the participants’ sociodemographic characteristics and the second was structured in nine attributes, being First contact, Access to the diagnosis, Access to treatment, Comprehensiveness of services, Longitudinality-relational, Coordination and Collaborative health actions, Family centeredness, Community orientation and Interpersonal communication. These domains have been proposed as assessment criteria to judge the quality of health care. The key definitions of the domains were based on the study developed by Haggerty et al.” |
| Data sources/ measurement | 8* | For each variable of interest, give sources of data and details of methods of assessment (measurement). Describe comparability of assessment methods if there is more than one group | 4/5 | *“*The study population consisted of 165 leprosy patients, identified through the Notifiable Diseases Information System (SINAN).”  “The data was obtained through the application of a questionnaire adapted from the PHC Assessment Tool (PCATool) [19] and validated by Villa and Ruffino-Neto (2009) [20] to study the performance of health systems in the control of chronic transmissible and neglected diseases, such as tuberculosis, HIV and leprosy. The authors also collected sociodemographic data and patient characteristics and the type of health services sought at the onset of signs and symptoms. Participants answered a tool using a five-item Likert response scale: never true (1), somewhat true (2), true half of the times (3), mostly true (4), always true (5).” |
| Bias | 9 | Describe any efforts to address potential sources of bias | 6 | “Two persons typed the data independently, after which both files were confronted to check for inconsistencies, using the software Statistica version 12.0.” |
| Study size | 10 | Explain how the study size was arrived at | 4 | “The study population consisted of 165 leprosy patients, identified through the Notifiable Diseases Information System (SINAN).” “…the authors considered patients diagnosed from 01 January 2009 to 31 December 2012.” |

Continued on next page

| Quantitative variables | 11 | Explain how quantitative variables were handled in the analyses. If applicable, describe which groupings were chosen and why | 5/19 | “Table 1 presents the key definitions of each domain investigated and the number of items each domain contains.” |
| --- | --- | --- | --- | --- |
| Statistical methods | 12 | (*a*) Describe all statistical methods, including those used to control for confounding | 6 | “Two persons typed the data independently, after which both files were confronted to check for inconsistencies, using the software Statistica version 12.0.  To assess the performance of the local health system to eliminate leprosy, the steps defined in other studies for performance assessment were followed [19].  Initially, univariate analysis was carried out with description of position (mean and median) and dispersion measures (standard deviation) of the study variables. Then, the mean score of each indicator in the tool was obtained, considering the sum of scores for each item divided by the number of participants. It also was computed a 95% confidence interval for the mean. The attributes were constructed based on the mean item scores. The following criteria were adopted: indicator below 3 unsatisfactory; between 3 and 4 regular; and 4 or more satisfactory.  Bivariate analyses were conducted, comparing leprosy patient groups with different level of incomes in relation to the attributes investigated. To stratify the groups regarding the income, the researchers considered the quartiles low, medium and high. Subsequently, the authors used one-way ANOVA and Kruskal-Wallis, the latter when the criteria of normality and homoscedasticity were not confirmed [22]. A two-sided p-value of ≤ 0.05 was defined as statistically significant.” |
|  |  | (*b*) Describe any methods used to examine subgroups and interactions | 6 | “Bivariate analyses were conducted, comparing leprosy patient groups with different level of incomes in relation to the attributes investigated. To stratify the groups regarding the income, the researchers considered the quartiles low, medium and high.” |
|  |  | (*c*) Explain how missing data were addressed | There were no missing data because the instrument was a Likert response scale |  |
|  |  | (*d*) *Cohort study*—If applicable, explain how loss to follow-up was addressed  *Case-control study*—If applicable, explain how matching of cases and controls was addressed  *Cross-sectional study*—If applicable, describe analytical methods taking account of sampling strategy | 4/5 | “The study population consisted of 165 leprosy patients, identified through the Notifiable Diseases Information System (SINAN). For the study, the authors considered patients diagnosed from 01 January 2009 to 31 December 2012. The inclusion criteria were: patient with an address in the urban area of Londrina and who were 18 years old or older. The authors considered as exclusion criteria: patients who lived in rural areas, corresponding to the regions of the city beyond the urban perimeter [18] or who were not found at home after three visits by the researchers.  Figure 1 shows the numbers of individuals in each study stage and the eligible and analyzed participants.” |
|  |  | (*e*) Describe any sensitivity analyses | Not used |  |
| Results | | | | |
| Participants | 13* | (a) Report numbers of individuals at each stage of study—eg numbers potentially eligible, examined for eligibility, confirmed eligible, included in the study, completing follow-up, and analysed | 6 | “One hundred and nineteen subjects participated in the study.”  “Twenty two percent of the patients (26) were classified as paucibacillary and 78.0% (93) as multibacillary” |
|  |  | (b) Give reasons for non-participation at each stage | 6 | “None of the subjects contacted refused to participate.” |
|  |  | (c) Consider use of a flow diagram | Not used |  |
| Descriptive data | 14* | (a) Give characteristics of study participants (eg demographic, clinical, social) and information on exposures and potential confounders | 6 | “Twenty two percent of the patients (26) were classified as paucibacillary and 78.0% (93) as multibacillary. Table 2 shows the participants’ sociodemographic characteristics. There is a balance among the sexes in terms of patients affected by leprosy. The participants’ ages varied between 42 and 65 years old and 66.4% (79) had finished primary school. In addition, 50.4% (69) patients were married and most of them gained between 1.2 and 3.3 minimum wages (MW) per month.  Considering the employment status, 47.1% (56) were employed, most of them self-employed; 30.2% (36) were retired and 9.2% (11) disability retired because of the disease. Regarding the patients’ housing conditions, most of them lived in their own house, made of concrete.” |
|  |  | (b) Indicate number of participants with missing data for each variable of interest | None, because the data was made through the questionnaire adapted from the PHC Assessment Tool (PCATool) and none of the subjects contacted refused to participate. |  |
|  |  | (c) *Cohort study*—Summarise follow-up time (eg, average and total amount) | - |  |
| Outcome data | 15* | *Cohort study*—Report numbers of outcome events or summary measures over time | - |  |
|  |  | *Case-control study—*Report numbers in each exposure category, or summary measures of exposure | - |  |
|  |  | *Cross-sectional study—*Report numbers of outcome events or summary measures | - |  |
| Main results | 16 | (*a*) Give unadjusted estimates and, if applicable, confounder-adjusted estimates and their precision (eg, 95% confidence interval). Make clear which confounders were adjusted for and why they were included | 21/22/23/24/25 | This item can be checked according to the results contained in the tables and observations at the end of the tables  **Table 3:** Performance of a local health system to eliminate leprosy according to the domains first contact, access to diagnosis and treatment, Londrina, State of Paraná, Brazil (2013).  **Table 4.** Performance of a local health system to eliminate leprosy according to the domains longitudinality-relational and interpersonal communication, Londrina, State of Paraná, Brazil (2013).  **Table 5.** Performance of a local health system to eliminate leprosy according to the domains comprehensiveness of services and Coordination and collaborative health actions, Londrina, State of Paraná, Brazil (2013).  **Table 6.** Performance of a local health system to eliminate leprosy according to the domains Family centeredness and Community orientation, Londrina, State of Paraná, Brazil (2013).  **Table 7.** Assessment of the domains by the leprosy patients according to their income stratus, Londrina, State of Paraná, Brazil (2013) |
|  |  | (*b*) Report category boundaries when continuous variables were categorized |  |  |
|  |  | (*c*) If relevant, consider translating estimates of relative risk into absolute risk for a meaningful time period |  |  |

Continued on next page

| Other analyses | 17 | Report other analyses done—eg analyses of subgroups and interactions, and sensitivity analyses | Not used |  |
| --- | --- | --- | --- | --- |
| Discussion | | | | |
| Key results | 18 | Summarise key results with reference to study objectives | 8/9 | “The study aimed to investigate how the patients perceive the system’s performance to eliminate leprosy. As observed, globally, this performance has not been satisfactory, as leprosy is diagnosed in a late stage and not close to the patient’s place of residence. It is highlighted that, although the patients turned to the Primary Health Care level when the symptoms started, that is not where the leprosy was diagnosed, which has entailed costs for the patients. At the specialized services where the patients were monitored, they satisfactorily assessed the relation with the health professionals at those services, but these are not articulated with the other care management and coordination services.” |
| Limitations | 19 | Discuss limitations of the study, taking into account sources of potential bias or imprecision. Discuss both direction and magnitude of any potential bias | 12 | The study limitations refer to the memory bias, in which many patients may not have remembered facts or occurrences, due to the time passed since the end of treatment. In addition, the use of an adapted instrument for leprosy should be highlighted, which may cause an information bias, and the number of losses, as many patients were not located due to a changed or non-existent address. |
| Interpretation | 20 | Give a cautious overall interpretation of results considering objectives, limitations, multiplicity of analyses, results from similar studies, and other relevant evidence | 12 | “Based on the results, it can be concluded that it is important to advance in the reorganization of the health services, to establish communication protocols among the different professionals working in these systems, and to invest in and value PHC, as investments at this care level come with a lower cost and, when well structured and with good problem-solving ability, they can promote a balance between the improvement of the population’s health and equity in the distribution of resources [34]. Using PHC as a partner can be an interesting measure to reduce the patients’ expenses and involve the community in the treatment of leprosy.” |
| Generalisability | 21 | Discuss the generalisability (external validity) of the study results | 11/12 | In addition, the study can serve as an important tool for managers to define local health policies for the elimination of leprosy and the reorganization of care services under the coordination of primary care professionals in the city under study. In line with some authors, a performance study should neither be an end in itself nor be forwarded as a strictly academic exercise, but should be focused on driving the development of health policies, strategies and programs, which was the direction taken in this study. |
| Other information | |  | | |
| Funding | 22 | Give the source of funding and the role of the funders for the present study and, if applicable, for the original study on which the present article is based |  | “This study was sponsored by Fundação de Amparo à Pesquisa do Estado de São Paulo (Fapesp)- PPSUS 2012/51235-5. The funders had no role in study design, data collection and analysis, decision to publish, or preparation of the manuscript.” |

*Give information separately for cases and controls in case-control studies and, if applicable, for exposed and unexposed groups in cohort and cross-sectional studies.

**Note:** An Explanation and Elaboration article discusses each checklist item and gives methodological background and published examples of transparent reporting. The STROBE checklist is best used in conjunction with this article (freely available on the Web sites of PLoS Medicine at http://www.plosmedicine.org/, Annals of Internal Medicine at http://www.annals.org/, and Epidemiology at http://www.epidem.com/). Information on the STROBE Initiative is available at www.strobe-statement.org.
